# Brain ageing changes proteoglycan sulfation, rendering perineuronal nets more inhibitory

**DOI:** 10.18632/aging.101256

**Published:** 2017-06-28

**Authors:** Simona Foscarin, Ruma Raha-Chowdhury, James W. Fawcett, Jessica C.F. Kwok

**Affiliations:** ^1^ John van Geest Centre for Brain Repair, Department of Clinical Neurosciences, University of Cambridge, Cambridge CB2 0PY, United Kingdom; ^2^ The Prague Centre of Reconstructive Neuroscience, Institute of Experimental Medicine AS CR, 14220 Prague 4, Czech Republic; ^3^ School of Biomedical Sciences, Faculty of Biological Sciences, University of Leeds, Leeds LS2 9JT, United Kingdom

**Keywords:** aging, perineuronal net, plasticity, glycosaminoglycans, sulfation

## Abstract

Chondroitin sulfate (CS) proteoglycans in perineuronal nets (PNNs) from the central nervous system (CNS) are involved in the control of plasticity and memory. Removing PNNs reactivates plasticity and restores memory in models of Alzheimer’s disease and ageing. Their actions depend on the glycosaminoglycan (GAG) chains of CS proteoglycans, which are mainly sulfated in the 4 (C4S) or 6 (C6S) positions. While C4S is inhibitory, C6S is more permissive to axon growth, regeneration and plasticity. C6S decreases during critical period closure. We asked whether there is a late change in CS-GAG sulfation associated with memory loss in aged rats. Immunohistochemistry revealed a progressive increase in C4S and decrease in C6S from 3 to 18 months. GAGs extracted from brain PNNs showed a large reduction in C6S at 12 and 18 months, increasing the C4S/C6S ratio. There was no significant change in mRNA levels of the chondroitin sulfotransferases. PNN GAGs were more inhibitory to axon growth than those from the diffuse extracellular matrix. The 18-month PNN GAGs were more inhibitory than 3-month PNN GAGs. We suggest that the change in PNN GAG sulfation in aged brains renders the PNNs more inhibitory, which lead to a decrease in plasticity and adversely affect memory.

## INTRODUCTION

Plasticity in the nervous system declines during ageing, as demonstrated by the progressive reduction in the ability of the central nervous system (CNS) to learn, to adapt to the changing environment and to compensate for damage. An outcome from this change in rodents is a progressive memory deficit detectable from around 12 months [1] (and Yang *et al*. unpublished observations). Earlier in life plasticity is at a high level during the postnatal critical periods, then many forms of plasticity are reduced [2–4]. One of the key factors underlying this closure of critical periods is the formation of perineuronal nets (PNNs) together with other changes in the extracellular matrix (ECM) environment [5, 6].

PNNs are specialized forms of ECM that condense around the surface of neurons at the end of the critical period, surrounding the synapses and blocking new synapse formation [6–10]. PNNs are composed of hyaluronan, tenascin, link proteins, and proteoglycans, the majority of which are chondroitin sulfate proteoglycans (CSPGs) [4, 11–13]. These consist of a core protein to which a number of chondroitin sulfate glycosaminoglycan chains (CS-GAGs) are attached. The most-studied type of PNN forms preferentially around parvalbumin fast-spiking GABAergic interneurons, where it acts to stabilize synaptic contacts, to limit further plasticity and to restrict the synaptic changes that underlie memory acquisition [4, 14, 15]. Various studies have shown that preventing PNN formation extends the critical period [5, 16] and removal of PNNs restores ocular dominance plasticity and other forms of plasticity back to the level seen during the critical period [6, 17]. More recently, it has also been demonstrated that the absence of PNNs increases terminal axonal sprouting, synaptic plasticity, and memory retention in adult mice and restores memory in an Alzheimer’s model [18, 19]. The mechanism by which PNNs control plasticity is still not clear; however, the observation that digestion of the CS-GAG chains with chondroitinase ABC (chABC) restores plasticity to the adult CNS clearly implicates the CS-GAGs as effectors of the PNNs. PNNs may exert their inhibitory functions through binding and presentation of active molecules such as semaphorin 3A and OTX2[7, 20, 21], through effects on the mobility of ion channels [21, 22], through modulating inhibitory connections onto parvalbumin interneurons [10] and through interactions between GAGs and the receptor PTPsigma [23]. CS-GAGs in PNNs constitute 2% of total brain CS-GAG, and the other 98% also surround synapses and also have effects on synapse dynamics [24].

The pattern of sulfation on CS-GAGs defines their charge structure, binding affinities and biological functions, and sulfation patterns change during development [25, 26]. Chondroitin-6-sulfate (C6S also known as CS-C) is the major form during early development, but its level decreases progressively during development, and again with the closure of critical periods. As C6S decreases, chondroitin-4-sulfate (C4S also known as CS-A) progressively increases during maturation and with the closure of critical periods, and is the predominant CS form in the adult nervous system [5, 25, 27, 28]. Thus the ratio between C4S and C6S increases during the period of PNN formation and critical period closure particularly in the PNN fraction of the extracellular matrix [5, 29]. C4S and C6S differ in their effects on axon growth and plasticity. C4S is inhibitory for axon growth and regeneration [27], whereas C6S is much less inhibitory to neurite growth and animals deficient in the main 6-sulfotransferase enzyme show greatly diminished plasticity and regeneration [30, 31]. High levels of C6S also inhibit PNN formation and restore ocular dominance plasticity in adult mice [31].

Given the role of CS-GAGs in controlling plasticity in the developing nervous system, and the observation that the absence of PNNs enhances memory persistence in adult mice, we hypothesized that the progressive decline of memory and plasticity during aging could be partly driven by changes in the PNNs, particularly by sulfation changes in the CS-GAGs in the CNS which make PNNs more inhibitory to new synapse formation. In order to test this hypothesis, we have isolated and analyzed GAGs from the PNNs and from the general diffuse ECM from rats ranging from 3 to 18 months of age. Our results show a reduction of C6S leading to a large increase in the C4S/C6S ratio in the PNNs in 12- and 18-month old brains. When extracted and tested for their ability to inhibit axon growth, the CS-GAGs from aged PNNs are more inhibitory to the growth of axons from adult dorsal root ganglia (DRG) than CS-GAGs from the PNNs of younger animals. The results provide the first evidence that the sulfation pattern of CS-GAGs in the PNNs changes with old age, and suggests that this change to more inhibitory PNNs might be a cause of memory deficits in old age. This is a new potential mechanism for memory loss in ageing, and is strongly supported by our current unpublished interventional work (Yang *et al*. unpublished observations).

## RESULTS

### Aged brains show an increase in C4S and a decrease in C6S

**Figure 1:**
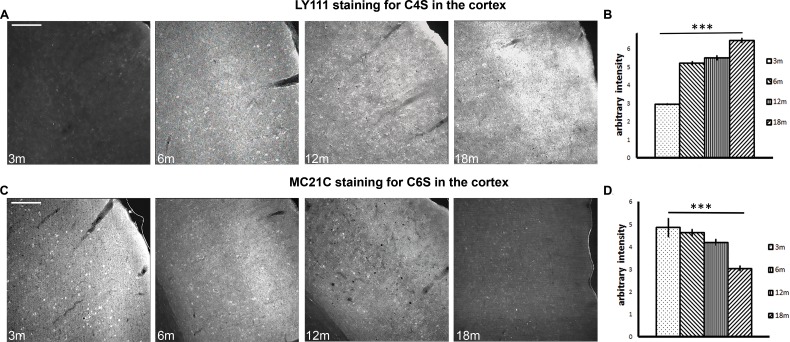
Brain immunohistochemistry shows a progressive increase in C4S and a progressive decrease in C6S from 3 to 18 months of age (**A**) Immunostaining of the auditory cortex with antibody LY111, which recognizes C4S, at time points 3 months to 18 months. (**B**) The histogram shows the quantification of the staining intensity of C4S from 3 to 18 months. (**C**) Immunostaining of the auditory cortex with antibody MC21C, which recognizes C6S, at time points 3 months to 18 months. (**D**) The histogram shows the quantification of the staining intensity of C6S from 3 to 18 months. Graphs show mean ± s.e.m. *** indicates a significant relationship between staining intensity and time by Kruskal Wallis, p >0.001. 3m, 3-month-old brains; 6m, 6-month-old brains; 12m, 12-month-old brains; 18m, 18-month-old brains; C4S, Chondroitin-4-sulfate; C6S, Chondroitin-6-sulfate. Scale bars: 200 μm.

To investigate the potential change in CS sulfation in aged brains, we first performed immunohistochemistry using antibodies targeting C4S and C6S on brain slices collected at different time points (Fig. 1). Brain slices of 3-month (3m), 6-month (6m), 12-month (12m) and 18-month old (18m) rats were stained with either LY111, an antibody which recognizes C4S GAG chains [32, 34, 37], or MC21C, an antibody which recognizes the C6S disaccharide motif on GAG chains [34, 35]. These antibodies bind preferentially to the differently sulfated CS-GAGs, but their exact binding site is not fully defined and their binding is only moderately specific, however they are currently the best available.

The staining intensity of brain sections was quantified by densitometry, with measurements taken from the auditory, visual and motor cortices. Staining intensity of C4S increased with age in the auditory cortex (representative images are shown in Fig. 1A), in the visual cortex, and in the motor cortex. Measurement of staining intensity in these areas showed a significant increase in C4S with ageing (Fig. 1B. 3m: 2.943 ± 0.069; 6m: 5.203 ± 0.118; 12m: 5.507± 0.147; 18m: 6.459± 0.138; Kruskal Wallis: p < 0.001. Data are averages of all the cortical areas analyzed). Conversely, the staining intensity of C6S decreased with age in the auditory cortex (representative images are shown in Fig. 1C), in the visual cortex, and in the motor cortex. Statistics done on cumulative averages of the areas showed a significant decrease in staining intensity (Fig. 1D. 3m = 4.848 ± 0.438; 6m = 4.628± 0.163; 12m = 4.186± 0.150; 18m = 3.034 ± 0.124; Kruskal Wallis: p < 0.001. Data are averages of all the cortical areas analyzed). This experiment suggests that the overall level of C4S increases in several brain regions during ageing, whereas C6S decreases.

### The reduction of C6S is specific to the PNNs from older animals

Immunohistochemistry showed a trend of C4S increase and C6S reduction in ageing brains. We needed to measure sulfation changes in the CNS with a more robust method, and to investigate if the change in CS sulfation is a general phenomenon in the brain matrix or is specifically associated with the PNNs. This was done by sulfation analysis from sequential extraction of GAGs from the different matrix compartments of rat brains using a method validated in our previous publications [36, 38]. GAGs from the general diffuse ECM are extracted in buffer 1 (B1, physiological saline), GAGs from membrane-associated matrix and cytoplasmic molecules are extracted in buffer 2 (B2, with detergent), charged matrix molecules are retained in buffer 3 (B3, 1 M sodium chloride), and GAGs from highly stable condensed matrix (~2% of total GAG in the brain) are finally retained in buffer 4 (B4, 6 M urea). The method provides an enrichment of PNN GAGs in B4 [38, 39]. GAGs isolated using this method were digested with chABC and the resulting disaccharides in fractions B1 and B4 were analyzed using fluorophore-assisted carbohydrate electrophoresis (FACE) for age-dependent changes in CS sulfation.

**Figure 2:**
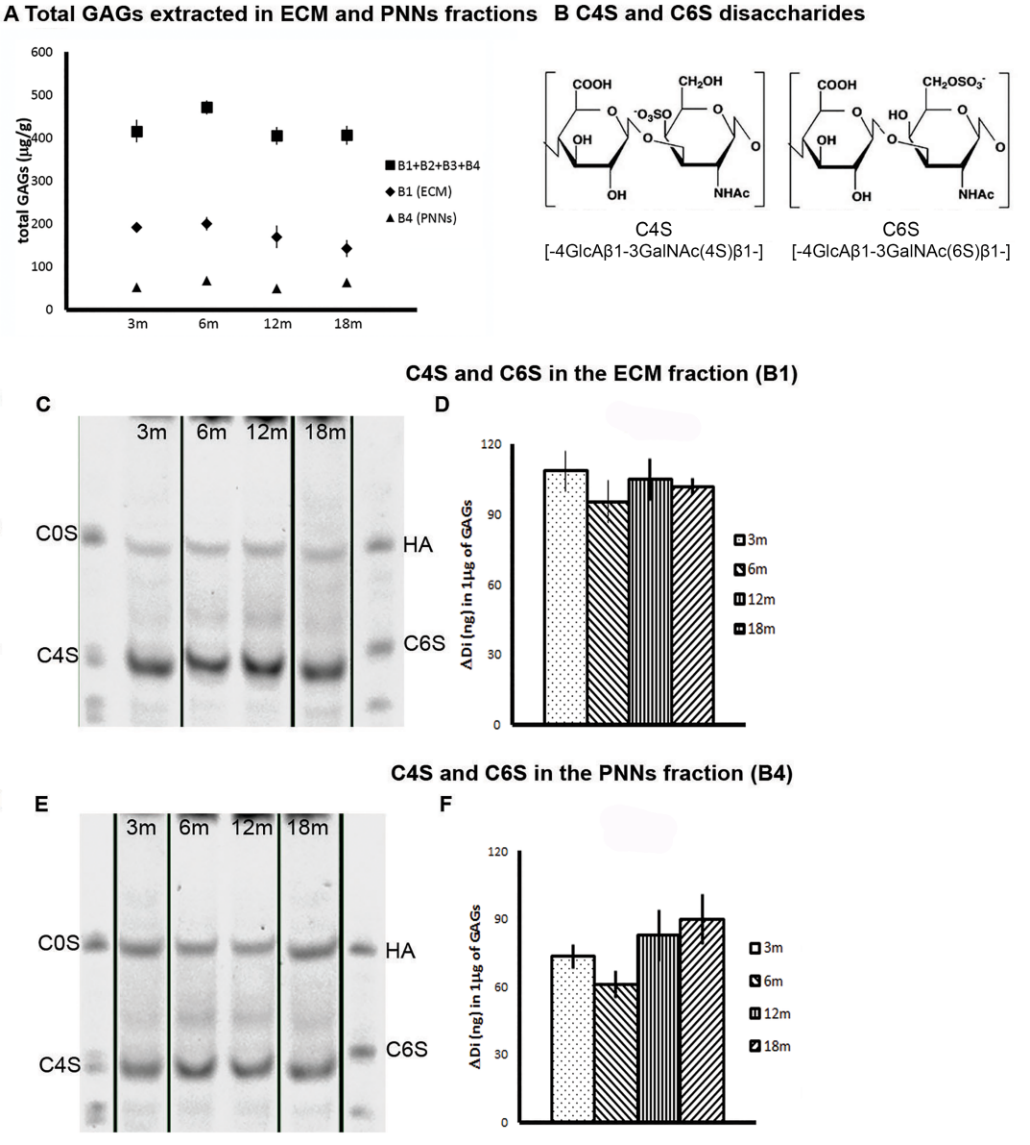
Biochemical analyses of CS-GAG sulfation. 12-, 18-month old brains shows an increase in the ratio C4S/C6S in the PNNs fraction (**A**) Measurement of the total CS GAGs quantity in the various fractions extracted from brains at time points from 3 to 18 months. There is no overall change in GAGs quantity with time. (**B**) The structure of the repeating disaccharide making up CS GAGs. The left diagram is sulfated in the 4 position (C4S), the right in the 6 position (C6S). (**C**) A composite image representing examples of FACE gels with CS GAGs extracted from the diffuse (B1) ECM at 3,6, 12 and 18 months. The GAGs were igested into disaccharides using chABC and the resulting disaccharides were electrophoresed in the FACE gel. The C4S disaccharides were identified by comparing the band location to the standard disaccharides in the first and last lanes. (**D**) Quantification of the FACE gels from the lower part of the sulfated band. C4S remains stable over time. (**E**) A composite image representing examples of FACE gels of CS GAGs extracted from the PNNs (B4) fraction at 3,6, 12 and 18 months. (**F**) There is a non-significant trend of an increase in C4S over time. Graphs show mean ± s.e.m. *** indicates a significant relationship between the considered CS GAGs and time by One Way Anova, p >0.001.** indicates a significant relationship between the considered ratio and time by Kruskal-Wallis test, p =0.002.B1, ECM fraction; B4, PNNs fraction; B1+B2+B3+B4, sum of GAGs from the four fractions of each brain; 3m, 3-month-old brains; 6m, 6-month-old brains; 12m, 12-month-old brains; 18m, 18-month-old brains; C4S, Chondroitin-4-sulfate; C6S, Chondroitin-6-sulfate; C0S, Chondroitin non-sulfated; HA, hyaluronic acid.

**Figure 3:**
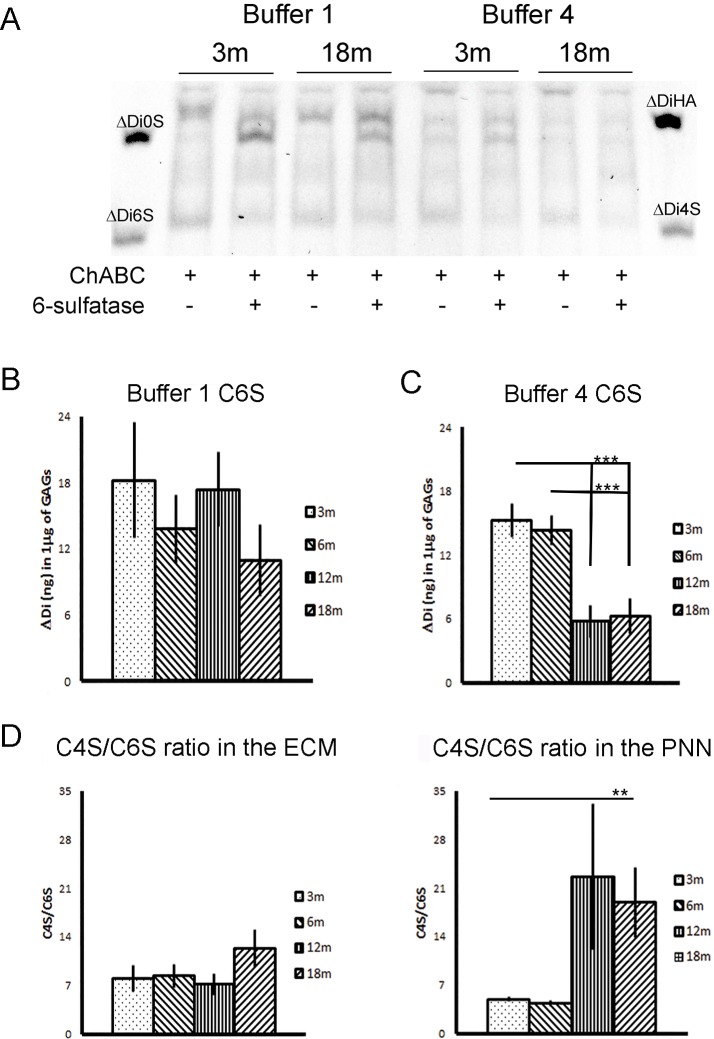
Quantification of C6S after chondro-6-sulfatase digestion (**A**) A composite picture taken from two gels, one with buffer 1 and the other with buffer 4. Each extract shows the ChABC digested lane and the same extracted digested with chondro-6-sulfatase. (**B** and **C**) Quantification of C6S levels in buffer 1 and buffer 4 extract. There is a significant reduction in C6S at 12m and 18m time points in buffer 4 fractions. (**D**) The ratio of C4S to C6S at the four time points, plotted respectively from the data in figure 2 and 3. The ratio significantly increases in favor of the inhibitory C4S in 12 and 18 months PNNs extracts. Graphs show mean ± s.e.m. *** indicates a significant relationship between the considered CS GAGs and time by One Way Anova, p >0.001.** indicates a significant relationship between the considered ratio and time by Kruskal-Wallis test, p =0.002.B1, ECM fraction; B4, PNNs fraction; B1+B2+B3+B4, sum of GAGs from the four fractions of each brain; 3m, 3-month-old brains; 6m, 6-month-old brains; 12m, 12-month-old brains; 18m, 18-month-old brains; C4S, Chondroitin-4-sulfate; C6S, Chondroitin-6-sulfate; C0S, Chondroitin non-sulfated; HA, hyaluronic acid.

We first asked whether the total GAG content isolated from the brain changes upon ageing. Fig. 2A shows that the total quantity of GAGs isolated from the general diffuse brain matrix (B1) and PNN matrix (B4) are stable; no age related changes were observed at any of the time points analyzed. We then proceeded to analyze the specific CS sulfation patterns, and in particular the level of C4S and C6S disaccharides (Fig. 2B) using FACE. The results confirm previous results showing that, in the mature brain, C4S is the major CS sulfation isoform (Fig.2, C-F) while C6S is present at a much lower level (Fig. 3, B-C). This observation is consistent for both GAGs isolated from the general ECM (Fig. 2, C and D; Fig. 3, A and B) and the PNN fraction (Fig. 2, E and F; Fig. 3, A and C). The sulfation pattern of the diffuse ECM fraction (B1) remained stable with ageing (Fig. 2D and 3B). The amount of C4S remained constant from 3 to 18 months (Fig. 2D. 3m: 108.623 ± 8.655; 6m: 95.448 ± 9.206; 12m: 104.970 ± 9.049; 18m: 101.968 ± 3.487); similarly, also the amount of C6S did not change significantly over time (the apparent decreasing trend was not significant) (Fig. 3B, right graph. 3m: 18.235 ± 5.218; 6m: 13.816 ± 3.107; 12m: 17.411 ± 3.407; 18m: 10.954 ± 3.211). In contrast, in the PNNs fraction (B4; Fig. 2E and F) the amount of C4S showed a non-significant tendency to increase with ageing (Fig. 2F. 3m: 73.306 ± 5.271; 6m: 60.955 ± 6.151; 12m: 82.715 ± 11.439; 18m: 89.721 ± 11.203); whereas we observed a reduction of C6S in the PNNs fraction from aged brains at 12 and 18 months (Fig. 3C. 3m: 15.256 ± 1.582; 6m: 14.364 ± 1.409; 12m: 5.749 ± 1.540; 18m: 6.252 ± 1.666; One Way ANOVA: p < 0.001; Bonferroni *post-hoc* comparisons significant for 3m vs 12m and 18m, and for 6m vs 12m and 18m). This constitutes a 60% reduction of C6S from young (3m and 6m) to old (12m and 18m) PNN GAG samples.

As effects on plasticity and regeneration are probably determined by the ratio of inhibitory C4S to permissive C6S, we looked at the ratio between C4S and C6S in the different fractions. In the diffuse ECM (B1), this ratio remained unchanged from 3 to 18 months (Fig. 3D, left graph. 3m: 7.996 ± 1.909; 6m: 8.371 ± 1.684; 12m: 7.160 ± 1.556; 18m: 12.336 ± 2.656). In contrast, in PNN extracts (B4), the ratio C4S/C6S increased significantly with age (Fig. 3D, right graph. 3m: 4.898 ± 0.335; 6m: 4.329 ± 0.400; 12m: 22.620 ± 10.540; 18m: 18.935 ± 5.112; Kruskal-Wallis: p = 0.002.). This means that between the young samples (3 m and 6 m) and the old samples (12 m and 18 m) the ratio C4S/C6S increased by more than 300%.

In conclusion, the biochemical results from glycan analysis show that the relative quantities of mono-sulfated disaccharides change in favor of the inhibitory C4S upon ageing, mainly due to a decrease in C6S at 12 and 18 months, and this change occurs specifically in the PNNs fraction. This change may make aged PNNs more inhibitory.

### The changes in PNNs sulfation pattern are not due to a change in overall gene expression

The most straightforward possibility to explain changes in the GAGs sulfation pattern would be a change in the expression of the genes regulating the synthesis of different sulfated groups. In mammals, C4S can be produced by three sulfotransferases isoforms, transcribed from *Chst11*, *Chst12*, and *Chst13* respectively [40, 41]. Formation of C6S is instead dependent on the sulfotransferases *Chst3* and *Chst7* [42, 43]. The mRNA levels of some of these enzymes change after brain injury [28]. We analyzed the expression of these genes in the somatosensory cortex of rats from 3 to 18 months of age to test whether gene regulation may account for the observed changes in PNNs sulfation.

**Figure 4:**
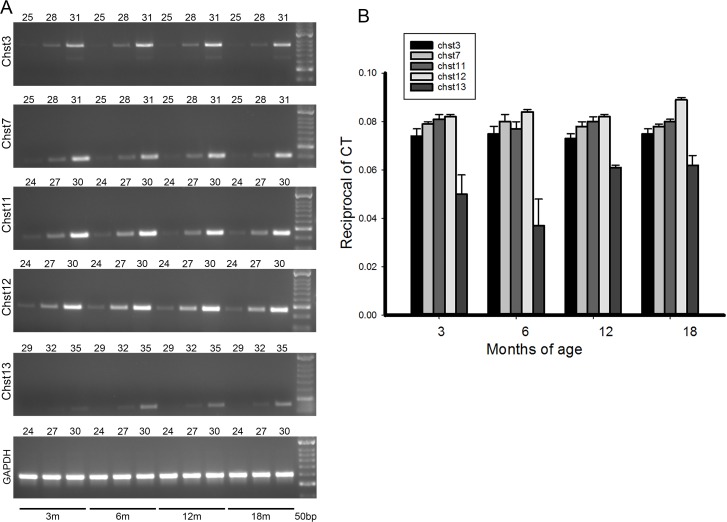
mRNA levels for the various chondroitin sulfotransferases do not change with ageing (**A**) shows typical PCR results for the various enzymes. The age of brain from which the mRNAs were extracted is shown by bars at the bottom. The number of cycles is shown by the number above each gel. (**B**) Quantification of the mRNA levels for the sulfotransferases at 4 age points. The method of quantification is described in the methods section. There are no changes of mRNA levels with ageing. Each bar represents results from at least 4 assays. Bars are SEM.

In the whole brain samples no changes in sulfotransferase mRNA expression with ageing were seen (Fig. 4). However, in our glycan sulfation measurements we saw changes in sulfation levels with age only in the PNN fraction, and this represents only 2% of total brain CSPGs. No sulfation changes were seen in the large majority of CSPGs making up the diffuse matrix. It is therefore not surprising that overall sulfotransferase mRNA levels did not change. It is possible that specific changes in sulfotransferase mRNA levels restricted to PV interneurons occur, but these changes would not have been detected by our assay.

### PNNs extracts from 18-month-old rats are more inhibitory for neurite growth than PNNs extracts from 3-month-old rats

**Figure 5:**
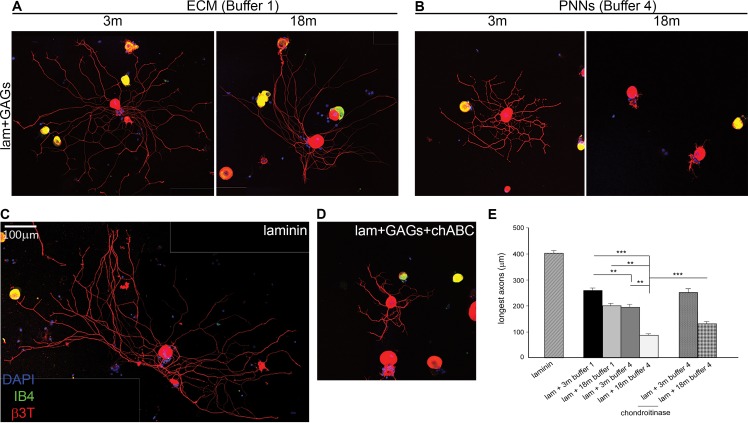
Axon growth *in vitro* is inhibited by GAGs, and more inhibited when GAGs are from PNNs fractions of old brains (**A**) Axon outgrowth from adult DRG neurons plated on laminin mixed with CS GAGs from diffuse ECM fraction (B1) of 3 or 18 month old brains. (**B**) Axon outgrowth from adult DRG neurons plated on laminin mixed with CS GAGs from PNN fraction (B4) of 3 or 18 month old brains. (**C**) Axon outgrowth from typical adult DRG neurons plated on laminin on laminin alone. (**D**) Axon outgrowth from adult DRG neurons plated on laminin mixed with CS GAGs from PNN fraction of 18 months brains (B4) and treated with chABC. (**E**) The graph shows average longest neurite of DRG grown 24 h on each plating condition. All the GAGs inhibit neurite outgrowth when compared with laminin alone; PNNs extracts are more inhibitory than ECM extracts; growth is further reduced when GAGs are from the PNNs fraction of aged brains; chABC treatment partially rescues the inhibitory effect of PNNs extracts from 18 months brains. Graphs show mean ± s.e.m. Asterisks indicate a significant relationship between average longest neurite and type of GAGs extract used for plating. One Way Anova and Dunnett post-hoc comparisons:***p >0.001, **p =0.002.B1, ECM fraction; B4, PNNs fraction; 3m, 3-month-old brains; 6m, 6-month-old brains; 12m, 12-month-old brains; 18m, 18-month-old brains; C4S, Chondroitin-4-sulfate; C6S, Chondroitin-6-sulfate; lam, laminin; chABC, chondroitinase ABC. Scale bar: 100 μm.

To assess whether the age-related change in CS sulfation in the PNNs would result in stronger inhibition to neurite outgrowth, we cultured adult DRG neurons on coverslips coated with the GAG extracts from different ages and fractions. This assay is a proven and sensitive method for assessing inhibition due to proteoglycans in general and CS in particular [44, 45]. The results were quantified by measuring the longest axon for each individual DRG neuron and averaging 120-180 neurons from three repeats of each condition. DRG neurons cultured on laminin alone showed extensive healthy neurite projections, with an average length of 402.105 ± 8.531 μm (Fig. 5C). All neurons cultured on isolated GAGs projected shorter neurites than when cultured on laminin, demonstrating the overall inhibitory nature of the isolated GAGs to neuronal growth (Fig.5E. One Way ANOVA and Dunnett *post-hoc*: p < 0.001 for every comparison). A comparison of the neurite length from DRGs cultured on different GAGs fractions of the same age showed that PNN extracts (B4; Fig. 5B) were always more inhibitory than diffuse ECM extracts (B1; Fig. 5A), with a more marked difference at the 18-month time-point, where PNN GAGs showed much more inhibition than at 3 months. Comparison between diffuse ECM (B1) and PNN (B4) GAGs from 3-month brains showed 22% less growth on PNN GAGs (Fig. 5: compare the left picture from panel A with the left picture from panel B; Fig. 5E. Average longest neurite on ECM: 256.221 ± 10.578μm; average longest neurite on PNNs: 199.193 ± 9.051 μm; Dunnett *post-hoc*: p = 0.002). Comparison between diffuse ECM (B1) and PNN (B4) GAGs from 18-month brains showed a difference of over 60% (Fig 5: compare the right picture from panel A with the right picture from panel B; Fig. 5E. Average longest neurite on ECM: 230.777 ± 12.568 μm; average longest neurite on PNNs: 84.386 ± 5.604 μm; Dunnett *post-hoc*: p < 0.001). Moreover, inhibition from the GAGs increased with age in the PNN fraction (B4; Fig. 5, B and E), but not in the diffuse ECM fraction (B1; Fig. 5, A and E). In the diffuse ECM assays (B1), axon growth was the same regardless the age of the extracts (Fig. 5: compare the two pictures in panel A; Fig. 5E. Average longest neurite on 3m B1 extracts: 256.221 ± 10.578 μm; average longest neurite on 18m B1 extracts: 230.777 ± 12.568 μm). However in the PNN extracts (B4), the inhibitory effect of 18-month extracts was 58% greater than that of the 3-month PNN extracts (Fig. 5: compare the two pictures in panel B; Fig. 5E. Average longest neurite on 3m B4: 199.193 ± 9.051 μm; average longest neurite on 18m B4: 84.386 ± 5.604 μm; Dunnett *post-hoc*: p < 0.001). To further confirm that the inhibition of axon growth was due to CS-GAGs, we performed a set of experiments treating the coverslips with the enzyme chondroitinase ABC (chABC) after GAG coating, in order to digest the CSs and remove their inhibition. We applied the enzymatic treatment on samples from the 3- and 18-month PNNs fraction (B4). We obtained a significant rescue of about 29% in 3-months and 57% in 18-months (Fig. 5: compare the right picture of panel B with the picture D; Fig. 5E. Average longest neurite on 3m B4 extracts: 196.812 ± 4.923 μm; average longest neurite on 3m B4 + chABC: 252.194 ± 7.461 μm; average longest neurite on 18m B4 extracts: 84.386 ± 5.604 μm; average longest neurite on 18m B4 + chABC: 132.356 ± 7.263 μm; Dunnett *post-hoc*: p = 0.001).

Overall, these results demonstrated that GAGs from the PNN extracts (B4) are considerably more inhibitory than those from the diffuse ECM (B1). Moreover, PNN GAGs become much more inhibitory with age, while diffuse ECM GAGs do not. This is a large effect for PNN GAGs, with DRG neurons grown on PNN fraction of 18-month-old brains having axons 80% shorter than when neurons are grown on laminin alone. This strong inhibitory effect is mainly due to the specific CSs present in the GAGs extracts, since eliminating CSs with chABC partially restores axon growth.

## DISCUSSION

Our hypothesis was that the decline in memory and plasticity in the ageing brain might be explained or correlated with changes in the brain ECM, particularly in the matrix composing PNNs. We have therefore examined ageing changes in the CS-GAGs because these sulfated chains are critically involved in determining the function of PNNs and in the control of memory and plasticity. Here, we demonstrate that CS-GAGs in the PNNs change with ageing, with a fall in C6S leading to an increase in the ratio between C4S and C6S. This change is functionally significant, because GAGs extracted from PNNs extracts become much more inhibitory to axon growth with ageing. Interestingly, there was no ageing change in the sulfation pattern of the CS-GAGs from the diffuse brain ECM, which makes up the great majority of brain CS-GAG. This diffuse ECM surrounds synapses and certainly plays a part in regulating their dynamics, but our findings reinforce the idea that PNNs have a specific regulatory function in plasticity and memory.

Old age is connected with a decline in many brain functions even in healthy individuals, with a predictable loss of memory function even in those who do not develop Alzheimer’s disease. The physiological mechanisms behind the decline in CNS function with ageing are still incompletely understood. Our previous work has demonstrated a link between CS-GAGs, PNNs, plasticity and memory [18, 19, 46]. Thus digesting brain CS-GAGs or removing PNNs can extend memory in normal rodents, or restore memory in models of Alzheimer’s disease and ageing [18, 19] (Yang *et al*. unpublished observations). A possible hypothesis for the loss of memory in ageing is therefore that there might be an event that changes the properties of PNNs in the aged brain. One way of reducing brain plasticity is to reduce the amount of 6-sulfated CS GAG; this was demonstrated by creating a knockout mouse lacking the Chst3 sulfotransferase, leading to an animal with much reduced plasticity and CNS axon regeneration [30]. Conversely, overexpression of Chst3 restores plasticity in the adult CNS [31]. Therefore, the results reported here are consistent the hypothesis that the ageing nervous system loses memory through the loss of C6S GAGs in PNNs.

PNNs around inhibitory GABAergic parvalbumin neurons are compact ECM structures composed of hyaluronic acid, tenascins, link proteins and proteoglycans, especially CSPGs [4, 11, 47]. These molecules are upregulated at the end of critical periods for plasticity and condense around the parvalbumin neurons, where they stabilize synapses and limit further plasticity [5, 6, 10, 48]. There are different aggrecan-based nets around other classes of neuron [49, 50]. Several studies have demonstrated a developmental regulation of C4S and C6S on total CS-GAGs extracts from the embryonic to early adult CNS. C4S is not only the most abundant disaccharide in the adult CNS, but also its relative quantity compared to C6S increases from embryos to adults. On the contrary, C6S is expressed at a high level at embryonic stages, but greatly downregulated in adulthood at the end of critical periods [5, 25, 28]. Interestingly, the resulting shift in the C4S/C6S ratio has recently been suggested as possible trigger of PNNs formation and critical period closure [29, 31]. Moreover, C4S has been shown to be more inhibitory than C6S [27, 30]. These known changes were an origin of our hypothesis that the relative quantities of C4S and C6S on the PNNs might change with ageing, thus rendering the environment of the aged brain progressively more inhibitory and less able to support memory and plasticity.

In order to study the relative quantities of C4S and C6S in the ageing rat brain, we first studied the distribution of C4S and C6S in the brains using immunohistochemistry. We decided to focus on three cortical areas in which PNNs formation clearly coincides with the end of the critical period and in which the formation of PNNs has been shown to inhibit subsequent plasticity, i.e. the motor cortex [51], the auditory cortex [52, 53] and the visual cortex [6]. In these cortical areas, our immunostaining results showed a consistent increase in C4S and a consistent decrease in C6S from 3 months to 18 months of age. However, the antibodies used in this study have partly-defined binding targets, and it is not known if they bind to the whole CS-GAG chain or just its terminal residues. We therefore did a biochemical analysis of sulfation of CS-GAGs in the ageing brain.

In previous work, we have asked whether the control of plasticity is due to CS-GAGs in the general diffuse ECM found throughout the brain, or whether it is the approximately 2% of CS-GAGs contained within PNNs that is responsible. A brain knockout of the PNN component Crtl1 link protein led to attenuated PNNs without any overall change in the quantity of brain CS-GAGs, yet these animals showed continuing plasticity with no closure of their critical periods [5]. This demonstrates that PNNs are the specific ECM structures involved in the regulation of this form of adult plasticity. Similarly, these animals with attenuated PNNs show a prolongation of object recognition memory, identically to animals in which all brain CS-GAG is digested with chABC [18]. These results focus attention on CS-GAGs in PNNs rather than in the diffuse matrix. It was therefore important in the present investigation to analyze GAGs modifications specifically in the PNNs as well as in the diffuse ECM. To this aim, we analyzed the CS GAGs isolated from the diffuse ECM and from the PNNs using the sequential extraction method [38, 39]. The procedure separates GAGs contained in different ECM structures: B1 –diffuse ECM, B4 – stable aggregated ECM including PNNs. In line with previous literature on adult GAGs, our results confirmed a higher level of C4S compared to C6S in the brain ECM [5, 28, 31, 38]. Our new result is that the levels of C4S and C6S in the PNNs continue to change upon ageing, with a very significant decrease in C6S and a tendency of increase for C4S. This leads to a large shift in the C4S/C6S ratio, particularly at 12 and 18 months of age in rats; at this same age mice show profound memory deficits [1] Yang et al., unpublished observations). However, these changes were restricted to the PNNs fraction and were not seen in the diffuse ECM. This result suggests that PNNs become more inhibitory in the aged brain due to an increase in the ratio of inhibitory C4S over the more permissive C6S.

Our biochemical results show no change of C4S during ageing while immunohistochemistry showed an increase in C4S. This discrepancy of results is likely due to the cross-interaction of the LY111 antibody to other types of chondroitin sulfations. The structural similarity of different sulfation forms are strikingly high and it is difficult to raise antibody to be specific to one form only. When other sulfation is present in high concentration, it is likely that the antibody will bind to these sulfation too. Unpublished data from the lab showed that LY-111 binds to CS-C, -D and -E at high concentration in dot blot assay.

To demonstrate directly that CS GAGs from PNNs become more inhibitory in the aged brain, we used a functional assay in which dissociated adult DRG neurons were grown on a mixed surface of laminin and CS-GAGs extracted from the brain at different ages. DRG neurons are very responsive to changes in extracellular environment, including the inhibition from CS proteoglycans [44, 45, 54]. We compared the growth of DRG neurites on GAG extracts from diffuse ECM (B1) and PNNs (B4) at the youngest (3m) and the oldest (18m) time points. GAGs from the PNNs were more inhibitory than GAGs from the diffuse ECM at both time points, supporting the hypothesis that PNNs are a specialized inhibitory structure of the ECM [5]. Importantly, the growth of DRG neurons was inhibited the most strongly by the PNN GAGs extracted from aged 18-month-old brains, while the inhibition was much less using PNN GAGs isolated from young rats. We suggest this age-related increase in inhibition is due to the loss of 6-sulfated GAGs in the PNN fraction. There was no change in 6-sulfation in the diffuse matrix and this did not show an age-related increase in inhibition of axon growth. Chondroitinase treatment partially rescued the growth of neurites from the old brains. This suggests that there are other glycan structures in the 18-month old GAG preparation which are conferring inhibition to DRG neurons. However, the result is sufficient to indicate and confirm that changes in CS sulfation confer a more inhibitory property to the aged PNNs.

Sulfation levels are determined by the activity of sulfotransferases, Chst11, Chst12, Chst13, Chst3, and Chst7 being the main 4 and 6- sulfotransferases. Several of these genes are regulated following CNS damage [28]. We measured mRNA levels for these enzymes in whole brain at different ages, finding no changes. This result probably reflects the absence of change in sulfation of the CS-GAGs in the diffuse ECM, which make up the great majority of the CS-GAG in the brain. It is possible that there were mRNA changes in the PV interneurons which produce 2% of CS-GAG that would not have been revealed by this assay. Although the changes in Chst expression are minimal, we have previously demonstrated that the small changes in sulfations are crucial in determining the binding properties of PNNs to semaphorin 3A and Otx2 [7, 20, 21].

In conclusion, our study demonstrates for the first time that PNNs become progressively more inhibitory during ageing, and that this is at least partly attributable to a shift in the degree of C4S and C6S sulfation. We hypothesize that these changes in PNNs lead to a loss of plasticity and memory in the aged brain, an idea strongly supported by our current work (Yang *et al*. unpublished observations). Modulation of PNN sulfation is therefore an interesting new target for restoration of memory in the aged brain.

## MATERIALS AND METHODS

### Animals

Sprague Dawley rats (Charles River) of 3-month-old (3m), 6-month-old (6m), 12-month-old (12m), 18-month-old (18m) were used for the experiments. Animals undergoing perfusion were anaesthetized with raising concentration of CO_2_ followed by terminal injection of Euthatal (1ml per 300g of weight). Collection of fresh tissues was performed after raising concentration of CO_2_ followed by cervical dislocation. All experiments were performed according to the “Animal (Scientific Procedures) Act 1986” and the “Guidance of the Operation of ASPA 2014”, and were approved by the Ethical Committee of the UK Home Office.

### Immunochemistry

Rats were perfused with 0.12 M phosphate buffer followed by 500 ml of 4% paraformaldehyde. Brains were dissected and post-fixed in the same fixative overnight at 4°C, then cryoprotected in 30% sucrose in 0.12 M phosphate buffer. 30-μm-thick coronal sections were cut using a sledge microtome and collected in 1X PBS. All incubations were done at room temperature in 1X PBS with 0.3% Triton X-100 and 0.02% sodium azide plus 10% donkey serum. Free-floating sections were incubated overnight with shaking in one of the following antibodies or binding proteins: biotinylated *Wisteria Floribunda* agglutinin (Sigma, 20 μg/ml), mouse anti-C4S IgM antibody (clone: LY111; Seikagaku, 1:500), mouse anti-C6S IgM antibody (clone: MC21C; Seikagaku, 1:500). After rinsing, slices were incubated for 1 h with biotinylated anti-mouse IgM antibody (Jackson Lab, 1:200) where needed and followed by 1 h incubation with Alexa Fluor 647-conjugated streptavidin (Invitrogen, 1:1000). All the sections were then rinsed and stained overnight with rabbit anti-parvalbumin antibody (Swant, 1:1000), followed by 1 h incubation with Alexa Fluor 568-conjugated anti-rabbit antibody (Invitrogen, 1:1000).

Fixed DRG cultures (on coverslips) were washed in 1X PBS with 0.3% Triton X-100 and 0.02% sodium azide, then incubated in PBS with 0.3% Triton X-100, 0.02% sodium azide and 5% normal donkey serum for 1 h. Coverslips were incubated for 2 h with rabbit anti-β3-tubulin antibody (Sigma, 1:400), mouse anti-CS IgM antibody (clone CS56; Sigma, 1:400), FITC-conjugate IB4 (Sigma, 1:200). After rinsing, the coverslips were incubated with secondary antibodies: biotinylated anti-mouse IgM (Jackson Lab, 1:250), Alexa Fluor 568-conjugated anti-rabbit antibody (1:400), Alexa Fluor 647-conjugated streptavidin (1:400), and counter stain with Hoechst 33342 (Roche; 1:10,000) for 1 h.

Slides or coverslips were mounted with FluoroSave (Calbiochem) and visualized with a Leica DM6000B epifluorescence microscope or with a Leica SPE1000 confocal microscope. Images were taken by keeping the parameters constant and analyzed with ImageJ.

### Densitometry

Previous literature has demonstrated that that LY111 stains specifically for C4S motif [32, 33] and that MC21C stains specifically for C6S motif [34, 35]. Densitometry analysis of LY111 and MC21C was performed in different areas in the cortex, including motor, visual and auditory cortices. 3 to 6 rats were used per time point. For each brain area, 3-8 images were taken at 10X magnification. Mean grey value data were quantified with ImageJ on an arbitrary scale 0-255, where 0 is a pixel with no signal and 255 is a pixel of the maximum intensity (i.e. saturated). For each picture, mean grey value of a stained area was normalized against mean grey value of an unstained area of the same image (background). Data are shown in graphs as normalized arbitrary intensities.

### GAG extraction

Biochemical analyses were performed on 5 animals at each age. Sequential extraction of GAGs from rat brains was performed following a modified protocol from [36]. Briefly, fresh frozen brains were first homogenized very gently in buffer 1 [B1: 50 mM Tris-buffered saline (TBS), 2 mM ethylenediamine tetraacetic acid (EDTA), and Complete Mini EDTA-free protease inhibitor cocktail (Roche)] using Potter-Elvehjem. Homogenates were centrifuged at 4°C and 23,000 xg for 20 min, supernatant collected, pellets resuspended in B1 and centrifuged again; supernatants from the two centrifugations were pooled. Subsequently, pellets were resuspended in buffer 2 (B2: 0.5% Triton X-100 in B1 plus protease inhibitor) and centrifuged twice, then in buffer 3 (B3: 1 M sodium chloride in B2 plus protease inhibitor) and centrifuged twice, then finally in buffer 4 (B4: 6 M urea in B2 plus protease inhibitor) and centrifuged twice. Supernatants from each buffer were dialyzed in 25 mM Tris-HCl and 5 mM EDTA pH 8.0 overnight at 4°C using 3.5K MWCO dialysis tubing (Thermo Scientific). Thereafter, samples were incubated overnight at 37°C with 200 mg/ml pronase (Roche). The following day, samples were centrifuged at 4°C and 23,000 xg for 20 min, supernatants were collected and proteins were precipitated with 5% trichloroacetic acid (TCA) for 1 h on ice. Samples were centrifuged at 4°C and 23,000 xg for 20 min, supernatants were collected and the pellets were washed with 5% TCA. Supernatants were then washed four times in 1:1 diethyl ether, which was subsequently evaporated overnight. Samples were then neutralized to pH 7.0 with 1 M sodium bicarbonate solution. The GAGs were finally precipitated with sodium acetate (final concentration of 5% w/v in the sample) and then with 4 volumes of 100% ethanol (EtOH) at 4°C overnight. Finally, samples were centrifuged at 2,000 xg and 4°C for 15 min, supernatants discarded, pellets containing GAGs were dried and resuspended in H_2_O.

GAGs quantification was performed using cetylpyridinium chloride (CPC) turbidimetry assay. Different dilutions of GAGs were prepared in a 96 well-plate, alongside with CS-A (Sigma, concentration ranging from 0.02 to 5 μg) acting as standard curves. CPC reagent was prepared fresh by mixing one volume of 0.2% CPC with one volume of 133 mM magnesium chloride. Absorbance was measured at 405 nm with the software Gene5. Concentration of GAGs in the samples was quantified against the obtained standard curves.

### Fluorophore-assisted carbohydrate electrophoresis (FACE)

GAGs extracted from each buffer in the previous step were digested into disaccharides with 0.1 U chABC (Sigma) in 100 mM ammonium acetate pH 8.0 for 2 h at 37°C, then precipitated in four volumes of EtOH for 16 h at 4°C. For C6S quantification, the samples were treated further with 50 mU of chondro-6-sulfatase (Seikagaku) for 1 hr. Subsequently, samples were centrifuged at 4°C and 18,000 xg for 5 min, and supernatants collected. The pellets were washed with EtOH and centrifuged for two more times. Supernatants from all the centrifuges were pooled and dried in a SpeedVac. The dried pellets were then derivatized with 2-aminoacridone and sodium cyanoborohydride for 16 h at 37°C.

0.5 μg of AMAC-conjugated disaccharide samples and standard disaccharides (5-20 ng of hyaluronic acid – HA, non-sulfated chondroitin - C0S, C4S, C6S; Seikagaku) were electrophoresed on a 30% polyacrylamide gel in Tris Glycine buffer (0.2 M Glycine, 2.5 mM EDTA, pH adjusted to 8.9 using Trizma Base) at 800 V until the dye front of phenol red exited the gel (30-40 min).

For C4S, gels were imaged in a UV chamber (UVitec). Bands size and intensity were quantified using ImageJ software. The intensity of the sample data was normalized against the intensity of non-sulfated CS (C0S) and the quantity of disaccharides was calculated using the standard curve of HA electrophoresed in the same gel. Data are represented as ng of disaccharides per μg of GAGs analyzed.

### Chondroitin sulfotransferase (Chst) gene expression

mRNA levels of the sulfotransferases involved in the formation of C4S and C6S were analyzed in 4 rats at ages 3,6,12 and 18 months. Brains were quickly dissected and frozen on dry ice. Subsequently, a piece of somatosensory cortex of about 4 mm side and 100 mg weight was dissected on ice and collected in ribolyser tubes (Fastprep-24, Mpbio) with 1 ml of TRIzol Reagent (Invitrogen). Samples were spun three times for 20 sec at speed 5.5 in a Fastprep-24 5G machine. After homogenization, 200 μl of chloroform were added and samples centrifuged for 15 min at 12,000 xg at 4°C. The aqueous phase was collected, another 200 μl of chloroform added, and centrifugation repeated. Samples were then diluted 1:1 in ice-cold isopropanol and centrifuged for 30 min at 4°C and maximum speed. The pellets were rinsed with EtOH 70% and dried briefly at room temperature before being resuspended in RNAse free water. The RNA thus obtained was treated with 20 U of DNAse-I (Roche) and 40 U of RNAse OUT (Invitrogen) in 3 M sodium acetate, pH4.0 and 0.5 M magnesium sulfate for 15 min at 37°C. RNA purification was performed with RNeasy Mini columns following manufacturer’s instructions (Qiagen). cDNA was synthesized from 1 μg of RNA by means of SuperScript III reverse transcription kit (Invitrogen), using Oligo(dT) primers and following the manufacturer’s protocol.

Gene expression was analyzed by means of semi-quantitative PCRs on samples of 300 ng of cDNA. The primers (Sigma) were either previously used [28] or newly designed to detect the expression of Chst11, Chst12, Chst13, responsible for the sulfation of C4S; and of Chst3, Chst7, responsible for the sulfation of C6S. Three PCRs with increasing number of cycles (cycle number selected to correspond to log phase of PCR reaction) were performed to quantify products of each set of primers. Glyceraldehyde-3-phosphate dehydrogenase (GADPH) was used as housekeeping gene. Primer sequences and details for each reaction are listed in Table 1. PCR products were electrophoresed for 1 h at 100 V (in a Scie-Plas chamber) on 1.5% agarose gel and were pictured in a UV chamber (UVitec) with constant parameters. Intensity of each picture was adjusted with Photoshop CS2 so that GAPDH bands were always saturated (255) and the bottoms of the wells were always black (0). Bands size and intensity were quantified with ImageJ software and normalized for the intensity of the 200 bp band of Hyperladder 50 bp (Bioline). Values obtained from the three increasing PCR of each sample were plotted on a logarithmic scale to calculate the exponential equation describing the expression of each gene. In every case, the cycle threshold (CT) was defined as the number of cycles needed to each sample for obtaining a band of intensity 2 on the logarithmic scale. For each gene, CTs calculated from animals of the same age were averaged.

**Table 1:** Sequences of PCR primers used to measure mRNA levels of sulfotransferases

Gene (enzyme)	Primers sequence	Cycles	Product size (bp)
Chst3 (CS6ST1)	(F) 5′-TTCGTKGGSGAGTTCTTCAAC-3′ (R) 5′-CTCATAGCGCACCARCATGT-3′	25-28-31	674
Chst7 (CS6ST2)	(F) 5′-CCGATAGGCCCTTCCACTTG-3′ (R) 5′-CAGTTGGCGTTGGTTTCCAG-3′	25-28-31	205
Chst11 (CS4ST1)	(F) 5′-AAGTTCGAGGAGTTCGTGGC-3′ (R) 5′-CATCTCGTCGGTAGTTCGGG-3′	24-27-30	237
Chst12 (CS4ST2)	(F) 5′-TGGGAGGAAGACTGGTTTGC-3′ (R) 5′-AGGTGCTTGGGTTGTGTCAT-3′	24-27-30	259
Chst13 (CS4ST3)	(F) 5′-GCGTCCCGCATTTGAAAACA-3′ (R) 5′-ACGTAGCAGTACAGAAGCCG-3′	29-32-35	246
GAPDH	(F) 5′-TTCCAGTATGACTCTACCC-3′ (R) 5′-ATGGACTGTGGTCATGAGCCC-3′	any	398

### Culture of adult dorsal root ganglion neurons

Coverslips were first coated overnight with poly-D-lysine (40 μg/ml) in PBS, then washed and coated with 2 μg/ml laminin and 25 μg/ml GAGs from the sequential extractions for 3 h. Some of the coverslips were subsequently treated with 0.1 U chABC (Sigma) for 2 h to digest the coated CS GAGs. Finally, coverslips were washed and preincubated at 37°C with medium (1% ITS, 1% PSF in DMEM; Gibco).

Sprague Dawley rats of 3 months were humanely sacrificed and DRGs were dissected. The dissected DRG were cut into smaller pieces and incubated in collagenase (0.2% in Ca^2+^-free HBSS; Sigma) for 90 min at 37°C, followed by the addition of trypsin (1 mg/ml; Sigma) at 37°C for 10 min. The DRG were then triturated, layered on 15% bovine serum albumin (BSA; Sigma) in medium (1% ITS, 1% PSF in DMEM) and centrifuged for 15 min at 1000 xg. The pellet containing DRG neurons was resuspended in culture medium (1% ITS, 1% PSF, 0.1 μg/ml NGF in DMEM) and centrifuged for 2 min at 2000 xg; supernatant discarded and pellet resuspended again. The dissociated DRG neurons were then cultured in the culture medium and kept in a 7% CO_2_ incubator. Cells were fixed for 10 min in warm 4% PFA at 24 h after plating, then kept at 4°C in PBS with 0.02% sodium azide until staining.

Different experimental conditions tested were: laminin alone, ECM extracts (B1 GAGs) from 3 m rats and 18 m rats, PNNs extracts (B4 GAGs) from 3 m rats and 18 m rats.

For the quantification of neurite length, neurons which are β3-tubulin positive and IB4 negative, and with cell diameter > 20 μm were imaged at 20X and the longest neurite per neuron was measured using ImageJ software. Data were averaged from three independent experiments and for each condition ~120-180 cells were measured.

### Statistical analyses

Data collected from animals of the same age were first tested for normal distribution and equal variance. If distributions were normal, data sets were compared by means of One Way ANOVA test, followed by Bonferroni or Dunnett *post-hoc* comparisons depending on homoscedasticity; the non-parametric Kruskal-Wallis test was used instead when distributions were not normal. For the *in vitro* assay, although distribution were not normal, the number of observations was high enough to justify parametric statistics. All the data analyses were performed by using Excel 2010 and SPSS 22. Unless otherwise specified, data are represented by means ± s.e.m. and tests results are considered significant when p < 0.05.
